# Use of the Modified Early Warning Score in intrahospital transfer of patients

**DOI:** 10.5935/0103-507X.20200074

**Published:** 2020

**Authors:** Luciele da Rocha Monzon, Márcio Manozzo Boniatti

**Affiliations:** 1 Universidade La Salle - Canoas (RS), Brazil.

**Keywords:** Emergencies, Patient transfer, Death, Continuity of patient care, MEWS, Emergências, Transferência de paciente, Óbito, Continuidade da assistência ao paciente, MEWS

## Abstract

**Objective:**

To verify whether there is an association between the Modified Early Warning Score before the transfer from the emergency room to the ward and death or admission to the intensive care unit within 30 days.

**Methods:**

This is a historical cohort study conducted in a high-complexity hospital in southern Brazil with patients who were transferred from the emergency room to the ward between January and June 2017. The following data were collected: sociodemographic variables; comorbidities, as determined by the Charlson index; reason for hospitalization; Modified Early Warning Score at the time of transfer; admission to the intensive care unit; care by the Rapid Response Team; mortality within 30 days; and hospital mortality.

**Results:**

A total of 278 patients were included in the study. Regarding the Modified Early Warning Score, patients who died within 30 days had a significantly higher score than surviving patients during this period (2.0 [1.0 - 3.0] versus 1.0 [1.0 - 2.0], respectively; p = 0.006). The areas under the receiver operating characteristic curve for death within 30 days and for ICU admission were 0.67 (0.55 - 0.80; p = 0.012) and 0.72 (0.59 - 0.84; p = 0.02), respectively, with a Modified Early Warning Score cutoff of ≥ 2. In the Cox regression, the Modified Early Warning Score was independently associated with mortality within 30 days after multivariate adjustment (hazard ratio 2.91; 95% confidence interval 1.04 - 8.13).

**Conclusion:**

The Modified Early Warning Score before intrahospital transfer from the emergency room to the ward is associated with admission to the intensive care unit and death within 30 days. The Modified Early Warning Score can be an important indicator for monitoring these patients and can prompt the receiving team to take specific actions.

## Abstract (pt)

**Objetivo:**

Verificar se há associação entre o *Modified Early Warning Score* antes da transferência da emergência para enfermaria e o óbito ou a admissão na unidade de terapia intensiva em 30 dias.

**Métodos:**

Trata-se de estudo de coorte histórica desenvolvido em hospital de alta complexidade do Sul do Brasil com pacientes transferidos da emergência para a enfermaria entre os meses de janeiro e junho de 2017. Foram coletados: variáveis sociodemográficas, comorbidades pelo índice de Charlson, motivo da internação hospitalar, pontuação do *Modified Early Warning Score* no momento da transferência, internação na unidade de terapia intensiva, atendimento pelo Time de Resposta Rápida, mortalidade em 30 dias e mortalidade hospitalar.

**Resultados:**

Foram incluídos 278 pacientes no estudo. Em relação ao *Modified Early Warning Score*, os pacientes com óbito em 30 dias apresentaram escore significativamente maior do que os pacientes sobreviventes nesse período (2,0 [1,0 - 3,0] versus 1,0 [1,0 - 2,0], respectivamente; p = 0,006). As áreas sob a curva Característica de Operação do Receptor para óbito em 30 dias e para admissão na UTI foram 0,67 (0,55 - 0,80; p = 0,012) e 0,72 (0,59 - 0,84; p = 0,02), respectivamente, com ponto de corte do *Modified Early Warning* Score ≥ 2. Na regressão de Cox, o *Modified Early Warning Score* apresentou associação independente com mortalidade em 30 dias, após ajuste multivariável (hazard ratio 2,91; intervalo de confiança de 95% 1,04 - 8,13).

**Conclusão:**

O *Modified Early Warning Score* antes da transferência intra-hospitalar da emergência para enfermaria está associado com admissão na unidade de terapia intensiva e óbito em 30 dias. Calcular o *Modified Early Warning Score* pode ser um indicador importante para acompanhamento desses pacientes, permitindo ações específicas da equipe receptora.

## INTRODUCTION

The recognition and timely treatment of patients with clinical deterioration are essential to improve their outcomes.^(1,2)^ In these situations, a screening score that identifies patients at risk of deterioration is necessary.

The Modified Early Warning Score (MEWS) is one of the tools used, especially upon admission to the emergency room and during ward hospitalization, to recognize patients at risk of clinical deterioration.^(3-6)^ The MEWS is composed of five physiological parameters: systolic blood pressure, heart rate, respiratory rate, axillary temperature, and level of consciousness. Any value outside the range considered normal receives a score between 1 and 3, and the total score ranges from 0 to 14.^(7,8)^ The initial validation of the MEWS was performed with 709 emergency patients and found that a MEWS ≥ 5 was associated with mortality and admission to the intensive care unit (ICU).^(7)^ Recent studies have shown high variability in the accuracy of this score, which has led to uncertainty regarding its use.^(2,4,9,10)^ Its role in the inter- or intrahospital transfer of patients is even more undefined. Although it has been applied in interhospital pretransfer,^(11-13)^ its use at the time of transfer between sectors of the same hospital has not yet been explored.

The objective of this study is to verify whether the MEWS can be used before intrahospital transfer to identify patients who may benefit from a higher level of care. Specifically, we assessed whether there was an association between the pretransfer MEWS and death or admission to the ICU within 30 days.

## METHODS

This was a historical cohort study conducted at the *Hospital de Clínicas de Porto Alegre*, a high-complexity public, general and university hospital affiliated with the *Universidade Federal do Rio Grande do Sul* (UFRGS), which is located in the city of Porto Alegre (RS, Brazil). The emergency department has 41 beds for clinical, gynecological, and surgical specialties. The hospital emergency department receives self-presenting cases and high-complexity referrals. Since June 2015, the service has applied the MEWS immediately before patients are transferred to the ward to identify clinical instability. Patients with scores ≥ 5 require medical evaluation to determine whether they can be transported or must remain in the emergency room until stabilization. Other patients are released for transfer.

The study was approved by the Research Ethics Committee of the *Hospital de Clínicas de Porto Alegre* (CAAE 72784817.3.0000.5327). Due to the observational and retrospective nature of the study, the consent form was waived.

Patients aged 18 years or older who were transferred from the emergency room to the ward between January and June 2017 were evaluated for eligibility. We selected 15% of the patients using a random number table; patients were distributed equally among the months of the study. Patients without a MEWS in the electronic medical record were excluded from the study.

The patients’ medical records were reviewed to collect the following data: sociodemographic variables; comorbidities, based on the Charlson index; reason for hospitalization; MEWS score at the time of transfer; ICU admission; care by the Rapid Response Team (RRT); mortality within 30 days; and hospital mortality. The medical records until hospital discharge were reviewed.

### Statistical analysis

Continuous variables are expressed as the mean ± standard deviation (SD) or as median and interquartile range (IQ), depending on the data distribution. Categorical variables are presented as absolute numbers and percentages. Depending on whether the data were normally or nonnormally distributed, Student’s *t*-test or Mann-Whitney test was used for continuous variables, and the chi-square test was used for categorical variables. The area under the receiver operating characteristic (ROC) curve was constructed to evaluate the discriminatory capacity of the MEWS, with ICU admission and death within 30 days as outcomes. The best cutoff point was defined by the sensitivity analysis, and specificity was determined using the Youden index. To adjust for potential confounders, *a priori* covariates were selected based on the clinical plausibility of the occurrence of the outcome. These covariates included age and Charlson index. Along with the MEWS, these variables were included in the Cox regression model. The assumption of proportionality of the risks was verified using the scaled Schoenfeld residuals. The level of significance was set at 0.05. Statistical analysis was performed using the Statistical Package for Social Sciences (SPSS) 22.0 (Chicago, IL, USA).

## RESULTS

During the study period, there were 2,002 transfers from the emergency room to the ward. A total of 15% (n = 300) of these patients were selected using the random numbers table; patients were distributed equally among the months of January and June 2017. Of these, 22 were excluded because they did not have a MEWS recorded in the medical records at the time of transfer, which resulted in 278 patients being included in the study. The scoring and the variables that comprise the MEWS are shown in table 1. The clinical and sociodemographic characteristics of the population are described in table 2. The main reason for hospital admission was infection. Only 3.6% of patients required transfer to the ICU after admission to the ward, and the mortality rate within 30 days was 6.8%. The mortality rates of patients who were and were not transferred to the ICU were 50% and 5.2%, respectively (p < 0.001). The mean age of nonsurvivors was significantly higher than that of survivors (65.9 ± 18.4 *versus* 56.7 ± 17.7; p = 0.029). There was no difference in age between the patients who were and were not transferred to the ICU (53.10 ± 25.6 *versus* 57.5 ± 17.6; p = 0.604).

**Table 1:** Variables and Modified Early Warning Scores

Variables	Scores						
	3	2	1	0	1	2	3
Heart rate (bpm)		≤ 40	41 - 50	51 - 100	101 - 110	111 - 120	> 120
Respiratory rate (rpm)		< 9		9 - 14	15 - 20	21 - 29	≥ 30
Systolic blood pressure (mmHg)	≤ 70	71 - 80	81 - 100	101 - 199		≥ 200	
Level of consciousness				Alert	Confused	Responsive to pain	Unconscious
Temperature (ºC)		≤ 35		35.1 - 37.8		< 37.8	

**Table 2:** Sociodemographic and clinical characteristics

Variable	Frequency (n = 278)
Sex, male	142 (51.1)
Age	57.3 ± 17.9
Reason for admission	
Respiratory	23 (8.3)
Cardiovascular	46 (16.5)
Gastrointestinal	48 (17.3)
Infection	66 (23.7)
Renal/metabolic	24 (8.6)
Neurological	25 (9.0)
Other	
Charlson index	1.0 (0 - 2.0)
MEWS	1.0 (1.0 - 2.0)
Distribution of MEWS	
0	9 (3.2)
1	139 (50.0)
2	85 (30.6)
3	34 (12.2)
4	10 (3.6)
5	1 (0.4)
ICU admission	10 (3.6)
Care by the RRT within the first 48 hours	9 (3.2)
Death within 30 days	19 (6.8)
Hospital death	24 (8.6)

MEWS - Modified Early Warning Score; ICU - intensive care unit; RRT - Rapid Response Team. The results are expressed as n (%), mean ± standard deviation or median (interquartile range).

Regarding the MEWS, patients who died within 30 days had a significantly higher score in the univariate analysis than did surviving patients (2.0 [1.0 - 3.0] *versus* 1.0 [1.0 - 2,0]; p = 0.006). The area under the ROC curve for death within 30 days was 0.67 (0.55 - 0.80; p = 0.012), and one analysis identified a MEWS cutoff point of ≥ 2 using the Youden index (sensitivity 73.7% and specificity 55.2%). The area under the ROC curve for ICU admission was 0.72 (0.59 - 0.84; p = 0.02), with a sensitivity of 90.0% and specificity of 54.9% for MEWS ≥ 2. When this cutoff point was used to evaluate the outcomes, the MEWS continued to show a significant association with death within 30 days and ICU admission (Table 3).

**Table 3:** Association of outcomes with pretransfer Modified Early Warning Score ≥ 2

Outcome	MEWS < 2 (n = 148)	MEWS ≥ 2 (n = 130)	OR (IC95%)	p value
Death within 30 days	5 (3.4)	14 (10.8)	3.4 (1.2 - 9.9)	0.015
ICU admission	1 (0.7)	9 (6.9)	10.9 (1.4 - 87.5)	0.007
Care by the RRT within 48 hours	3 (2.0)	6 (4.6)	2.3 (0.6 - 9.5)	0.31

MEWS - Modified Early Warning Score; OR - odds ratio; 95%CI - 95% confidence interval. Odds ratio for Modified Early Warning Score ≥ 2 versus Modified Early Warning Score < 2, analyzed with univariate logistic regression.

**Figure 1:**
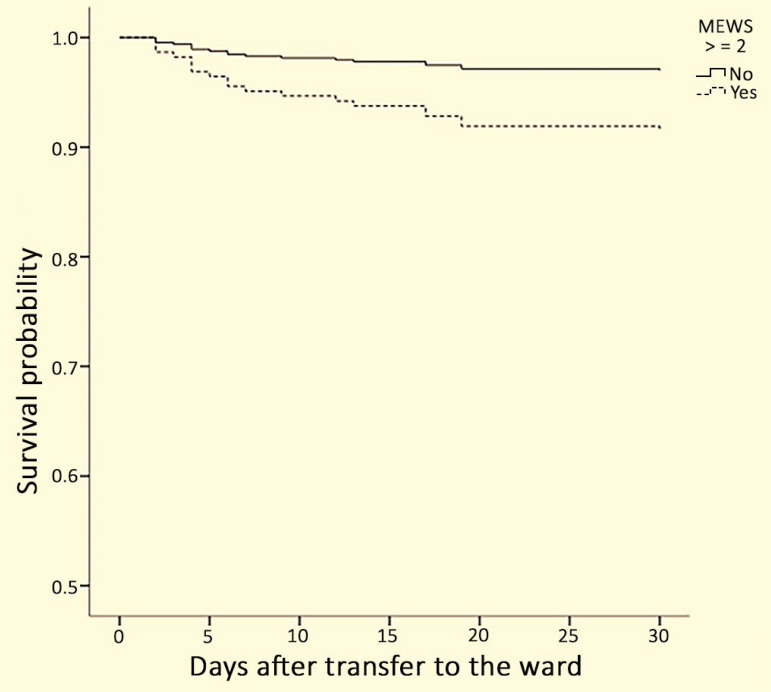
Cox regression showing 30-day survival according to the Modified Early Warning Score adjusted for age and Charlson index. MEWS - Modified Early Warning Score.

In the Cox regression, the MEWS showed an independent association with mortality within 30 days after multivariate adjustment (Table 4 and Figure 1).

**Table 4:** Cox regression model for mortality within 30 days

Variable	HR	95%CI	p value
Age	1.028	1.000 - 1.057	0.050
Charlson index	1.176	0.986 - 1.402	0.071
MEWS	2.910	1.042 - 8.129	0.041

HR - hazard ratio; 95%CI - 95% confidence interval; MEWS - Modified Early Warning Score.

## DISCUSSION

We found that the MEWS, when determined immediately before the transfer of patients from the emergency room to the ward, is independently associated with an increased risk of ICU admission and death within 30 days. Although the MEWS has already shown an association with these outcomes in other scenarios, this is the first study that demonstrates this association before intrahospital transfer (after initial emergency care) and in lower-risk patients (patients with a score ≥ 5 usually are not transferred to the ward at the study site).

Several studies have evaluated the use of the MEWS as a tool for predicting mortality, with the area under the ROC curve ranging from approximately 0.70 to 0.89 for the most frequently used cutoff point (MEWS = 5) and specificity and sensitivity ranging from 0.67 to 0.72 and 0.65 to 0.71, respectively.^(3,14-16)^ In the transport scenario specifically, the association of the MEWS with worse outcomes has already been demonstrated for the transfer of patients between institutions.^(11-13)^ Its use in intrahospital transport has not yet been explored. In this scenario, in addition to determining the risk associated with transport, the MEWS can serve as a screening tool to identify patients at risk of clinical deterioration who may benefit from specific actions in the receiving unit (greater monitoring, more frequent reevaluations, preemptive RRT evaluation, etc.).

There is some consensus that a MEWS of 5 or more is associated with imminent clinical instability; however, a lower cutoff point may be necessary for specific populations with lower risk.^(17,18)^ In our study, the cutoff point of 2 showed the best accuracy and moderate discriminatory power, especially for ICU admission. Another study that evaluated the accuracy of the MEWS before interinstitutional transportation also found a lower cutoff point (= 1).^(11)^

Regarding the RRT care in the first 48 hours, we found no association with MEWS. A recent study also found no association between MEWS and clinical deterioration in the first 48 hours of admission, defined by the need for RRT care.^(19)^

This study has some limitations. The study was conducted at only one center, which limits the generalizability of the results. In addition, this was a retrospective study, with the biases inherent to this type of design. Finally, the MEWS was evaluated only once. Dynamic changes in the score could provide better accuracy.

## CONCLUSION

The Modified Early Warning Score before intrahospital transfer from the emergency room to the ward was associated with admission to the intensive care unit and death within 30 days. As patients with Modified Early Warning Score ≥ 2 present higher mortality and a higher rate of admission to the intensive care unit in this scenario, the Modified Early Warning Score may be an important indicator for patient monitoring and informing specific actions by the receiving team. New prospective multicenter studies are needed to validate these findings.
